# Preliminary Clinical Outcomes of the Hello Sunday Morning Alcohol and Wellbeing Self-Assessment: Feasibility and Acceptability Study

**DOI:** 10.2196/48245

**Published:** 2023-10-24

**Authors:** Kathryn Fletcher, Alex Moran-Pryor, Dominique Robert-Hendren

**Affiliations:** 1 Hello Sunday Morning Sydney Australia; 2 Centre for Mental Health Swinburne University of Technology Melbourne Australia

**Keywords:** web-based, screening, alcohol use, brief intervention, well-being, psychological distress, digital health

## Abstract

**Background:**

Alcohol-related injuries and diseases are a leading cause of morbidity and mortality worldwide. Early intervention is essential given the chronic, relapsing nature of alcohol use disorders. There is significant potential for widely accessible web-based screening tools to help individuals determine where they stand in terms of alcohol use and provide support recommendations. Screening and brief interventions (SBIs) provide individuals with a stigma-free opportunity to learn and think about the potential risks of drinking and prompt help-seeking behavior by incorporating behavior change techniques. Furthermore, as excessive alcohol use and mental health problems often occur concurrently, SBIs for both conditions simultaneously can potentially address a critical gap in alcohol and mental health treatment.

**Objective:**

We investigated the feasibility, acceptability, and clinical outcomes of participants completing the Alcohol and Wellbeing Self-assessment (A&WS), a web-based SBI.

**Methods:**

The A&WS is freely available on the Hello Sunday Morning website as part of an uncontrolled observational prospective study. Feasibility was assessed based on the number of respondents who commenced and subsequently completed the A&WS. Acceptability was measured via participant feedback to determine overall satisfaction, perceived helpfulness, and likelihood of recommending the A&WS to others. Clinical outcomes were measured in two ways: (1) self-reported changes in alcohol consumption (Alcohol Use Disorders Identification Test score) or psychological distress (Kessler Psychological Distress Scale score) over time and (2) help seeking—both self-reported and immediate web-based help seeking. Preliminary baseline data collected for the first 9 months (March 2022 to December 2022) of the study were reported, including the 3-month follow-up outcomes.

**Results:**

A total of 17,628 participants commenced the A&WS, and of these, 14,419 (81.8%) completed it. Of those 14,419 who completed the A&WS, 1323 (9.18%) agreed to participate in the follow-up research. Acceptability was high, with 78.46% (1038/1323) reporting high satisfaction levels overall; 95.62% (1265/1323) found the A&WS easy to use and would recommend the tool to others. The 1-, 2-, and 3-month follow-ups were completed by 28.57% (378/1323), 21.09% (279/1323), and 17.61% (233/1323) of the participants, respectively. Significant reductions in the Alcohol Use Disorders Identification Test Consumption subscale (*P*<.001) and Kessler Psychological Distress Scale scores (*P*<.001) were observed over the 3-month follow-up period.

**Conclusions:**

Our results suggest that the A&WS is a highly feasible and acceptable digital SBI that may support individuals in making changes to their alcohol consumption and improve their psychological well-being. In the absence of a control group, positive clinical outcomes cannot be attributed to the A&WS, which should now be subjected to a randomized controlled trial. This scalable, freely available tool has the potential to reach a large number of adults who might not otherwise access help while complementing the alcohol and mental health treatment ecosystem.

## Introduction

### Background

Despite being preventable, alcohol-related injuries and diseases are a major cause of morbidity and mortality [1]. Worldwide, alcohol use contributed to 3 million deaths and 132.6 million disability-adjusted life years in 2016 [2]. Problematic alcohol use is a global issue, with almost 40% of those consuming alcohol reporting heavy episodic drinking (World Health Organization [2]). In 2019, a total of 1 in 4 people in Australia drank at a risky level at least monthly, whereas 1 in 6 exceeded the lifetime risk guidelines [3]. The COVID-19 pandemic exacerbated alcohol use, with 20% of respondents to an Australian National University study reporting increased alcohol use. Gender and age differences were observed, with women aged 30 to 40 years and men aged 20 to 30 years increasing their alcohol use. Furthermore, for both men and women—but particularly for men—psychological distress in 2020 was strongly associated with higher self-reported increases in alcohol consumption since the spread of COVID-19 [4]. In Australia alone, the cost of alcohol-related harms was estimated at Aus $22.6 billion (US $14.4 billion) in 2021 [5], whereas the problem of secondary harms (alcohol-related harms to family, friends, and others) was estimated at Aus $19.8 billion (US $12.6 billion) [6].

Early intervention is essential given the chronic, relapsing nature of alcohol use disorders. There is significant potential to close the gap between diagnosis and treatment seeking using widely accessible screening tools to help individuals determine where they stand in terms of alcohol use, with recommendations on which services to access. Most Australians see a general practitioner (GP) at least annually [7], positioning the primary care context as a useful portal to screen for alcohol use issues. However, significant variability exists in terms of the frequency with which physicians screen for patient alcohol consumption (6%-77% of patients) [8-10]. A key barrier to screening for alcohol consumption is time; many clinicians are time poor, with consultations focusing on the presenting issue. In addition, in Australia, <20% of those with problematic drinking seek treatment for their alcohol use [11], with the median time to first treatment for someone experiencing an addiction to alcohol being 18 years [12]. Even when presenting for treatment, individuals are hesitant to discuss their alcohol use. This is also the case internationally. As overviewed by Tansil et al [13], one study found that only 16% of adults in the United States and 25% of adult binge drinkers reported ever discussing alcohol use with a health professional, showing little change since 1997. Therefore, screening services need to be easily accessible, acting as a low-intensity touch point.

### Technology-Based Screening

Technology-based screening is a viable option to reduce practical barriers to help seeking. Along with being highly accessible and cost-effective, this type of screening is preferable for those with problematic alcohol use because of the anonymity it provides given the stigma associated with disclosure [14,15].

Screening and brief interventions (SBIs) are evidence-based interventions for a wide range of substance use disorders, including alcohol use disorders [16]. They can be delivered entirely digitally and are sometimes referred to as electronic SBIs (e-SBIs). SBIs focus on identifying individuals who drink at levels that have the potential to negatively affect their health and motivate those at risk to change their behavior [17]. Importantly, SBIs complement but do not replace treatment services for alcohol dependence. In essence, these interventions provide individuals with an opportunity to learn and think about the potential risks of drinking and decide what they want to do.

SBIs generally contain two key elements: (1) screening individuals for excessive drinking and (2) delivering a brief intervention that provides personalized feedback on the risks and consequences of excessive drinking [13]. The brief intervention component can be extended to include (3) motivational feedback (low level includes general advice on how to reduce excessive alcohol consumption, and high level includes more individually tailored messages based on factors such as readiness to change or developing personal goals) and (4) normative feedback comparing an individual’s own alcohol consumption with that of others (eg, gender and age norms).

As overviewed by White et al [18], several studies on problematic drinkers confirm the acceptability of e-SBIs for alcohol use, and use data confirm that they are accessed by numbers of users that would overwhelm traditional face-to-face services. In Australia, a pilot study conducted by Turning Point and Monash University [19] examined the uptake of web-based screening for alcohol and substances. Acceptability was high, with all participants reporting the screening as helpful and providing a positive experience in clarifying further help options. Almost half (47.8%) indicated that they would seek further professional support after completing the screening, leading the authors to conclude that this type of self-screening can act as a bridge to appropriate treatment as well as support self-help and natural recovery for those unlikely to present to face-to-face services.

### Does Technology-Based Screening Work?

There is evidence that e-SBIs can reduce alcohol consumption [18,20]. An earlier systematic review of digital interventions for alcohol use found that consumers can benefit from them and that they may be particularly useful for groups less likely to access traditional alcohol-related services, such as women, young people, and at-risk users [18]. Overall, web-based alcohol interventions (whether only involving brief personalized feedback or comprising multiple modules) brought about small but meaningful reductions in alcohol consumption, blood alcohol concentration, and a range of other alcohol-related measures. They appeared to be more efficacious than assessments alone or general education about alcohol. Donoghue et al [21] conducted a systematic review and meta-analysis to determine the effectiveness of e-SBIs over time in non–treatment-seeking hazardous and harmful drinkers. The findings indicated that e-SBIs contributed to significant reductions in weekly alcohol consumption between 3 and <12 months of follow-up. A later systematic review of randomized controlled trials (RCTs) primarily focusing on testing e-SBIs with differing levels of intervention (eg, normative vs motivational feedback) found that those who received e-SBIs consistently reported greater reductions in excessive alcohol consumption than controls [13]. The impact on excessive drinking was most pronounced for measures of binge drinking frequency and less pronounced for average consumption. Across the studies, a median reduction of 23.9% in binge drinking intensity (maximum drinks/episode) was quantified, along with a 16.5% reduction in binge drinking frequency. Reductions in drinking measures were sustained for up to 12 months. The effects of e-SBI on measures of alcohol-related harms were less pronounced, but alcohol-related harms decreased overall.

However, problematic alcohol use does not occur in isolation. Mental health problems—most commonly depression and anxiety—generally occur concurrently [22] and are associated with adverse outcomes [23,24]. Furthermore, worldwide, mental health worsened during the COVID-19 pandemic [25]. Therefore, screening for common co-occurring conditions with alcohol use is essential not only to increase awareness but also to provide holistic care, particularly given evidence that concurrent treatment of mental health problems (eg, depression) and problem drinking is more effective than treating either condition alone [26].  Given that mental health conditions can be missed during routine care, with only half of patients recognized by their GPs as having one [27], formal screening tools have the potential to fill a critical gap.

As overviewed by Choi et al [28], there is mixed evidence on the effectiveness of web-based screening for mental health issues. Several studies have found that web-based mental health screening with personal feedback can serve as a strategy to engage consumers. For example, Gill et al [29] found that one-third of participants came back to complete one or more follow-ups after initial screening and feedback for depression. Uncontrolled studies have found that this approach can facilitate help seeking. For example, Kim et al [30] found that 42% of university students who received positive screening results after using a self-help mental health screening website requested a referral to the university’s mental health clinic. Similarly, BinDhim et al [31] provided personal score feedback in a depression-screening app, recommending that those with scores above the threshold seek help from a health care professional. Approximately 38% of users who did not have a previous self-reported depression diagnosis indicated that they had consulted a health care professional after 1 month. In contrast, the first RCT [32] investigating the impact of web-based screening on depression and anxiety in a community sample found differing results. Screening was administered along with tailored feedback, including resource and service recommendations, and participants were followed up 3 months later. The findings suggested that providing tailored feedback based on web-based screening may be ineffective for promoting professional service use or for mental health outcomes. However, 2 nuances are worth noting. First, the way in which personal feedback is presented influences behavior in different ways [28]. User-friendly, comprehensible information may be more useful than simply providing score feedback as it is personally relevant and may strengthen motivation for behavior change [33]. Furthermore, a normative comparison of an individual’s results with those of a reference group may increase the salience of the feedback. Second, the way in which outcomes are measured following screening and feedback requires consideration. Choi et al [28] highlighted that most studies to date have only focused on seeking professional help as an outcome of screening and feedback. An alternative focus could be on immediate web-based help seeking, which reduces loss to follow-up and may be more meaningful than face-to-face help seeking, at least in the short term. Interestingly, web-based help-seeking rates in one study ranged from 26% to 60% [28], comparable with the rates of seeking face-to-face help following web-based screening found in other studies.

### This Study

The Alcohol and Wellbeing Self-assessment (A&WS) was designed using a theory-based procedure in which validated measures of alcohol consumption, psychological distress, and readiness to change were combined with behavior change techniques (BCTs) that have shown some evidence of effectiveness (either alone or in combination) in other e-SBIs. On the basis of the BCT taxonomy developed by Michie et al [34] and nudge techniques (BCTs developed within the field of behavioral economics that preserve choice while guiding individuals toward behavior with population-level benefits) [35], the A&WS provides feedback in the form of a Personal Snapshot Report comprising (1) *social comparison* (personalized normative feedback on alcohol consumption and psychological distress scores relative to peers [age and gender]), (2) *information about consequences (health and emotional) from a credible source* (information about levels or patterns of drinking and health risks or other negative consequences based on the current drinking level and advice about national guidelines for alcohol consumption [country specific where possible]), and (3) *social support* (*practical;* provision of links to information and services to help reduce alcohol consumption and improve psychological well-being).

Additional components included feedback on readiness to change (with motivational interviewing language to prompt individuals to start thinking about how to change their relationship with alcohol); psychoeducation (brief information about harm reduction strategies for alcohol consumption); and, for those who agreed to participate in the follow-up research component, brief monthly follow-ups with feedback on changes in alcohol consumption and psychological distress.

### Objectives

The study aims were to investigate the feasibility, acceptability, and clinical outcomes of participants completing the A&WS. Feasibility was defined in terms of uptake, measured via the number of respondents who commenced the A&WS who subsequently completed the tool. Acceptability was measured via feedback from participants in terms of their overall satisfaction, perceived helpfulness, and likelihood of recommending the A&WS to others. Clinical outcomes were assessed in two ways: (1) changes in alcohol consumption (Alcohol Use Disorders Identification Test [AUDIT] score) or psychological distress (Kessler Psychological Distress Scale [K-10] score) over time and (2) help seeking—both self-reported and immediate web-based help seeking as quantified by clicking on hyperlinks in the Personal Snapshot Report (ie, suggested services and resources and “Information Packs” on alcohol-related issues). We hypothesized that participants who completed the A&WS would report reduced AUDIT and K-10 scores over time and engage in help seeking.

## Methods

### Design

The A&WS was made freely available on the Hello Sunday Morning website [36] as part of an uncontrolled observational prospective study. Minimal marketing and advertising were undertaken, including social media posts (Facebook, Instagram, Twitter, and LinkedIn) and posts on the Hello Sunday Morning newsletter. Following completion of the A&WS, those who agreed to participate in the follow-up research component were asked to complete a feedback questionnaire (detailed in the following sections) and a series of follow-ups (1, 2, 3, 4, 5, 6, and 12 mo) to track their alcohol consumption and psychological distress over time. Follow-up survey links were emailed to participants, and a reminder was sent if they were not completed within 1 week. To assist with reducing attrition, participants were entered into a draw to receive an Aus $150 (US $95.56) Amazon voucher if they completed the 3-, 6-, or 12-month follow-ups. All questionnaires were administered on the web using Qualtrics (Qualtrics International Inc).

This paper reports preliminary baseline data collected for the first 9 months (March 2022 to December 2022) of the study, including the 3-month follow-up outcomes.

### Participants

Website visitors were invited to complete the A&WS. Other than being aged ≥18 years, no exclusion criteria were applied to ensure a real-world representative sample.

### Intervention

The A&WS comprises basic demographic questions (age, gender, and country of residence) followed by validated questionnaires assessing baseline (1) alcohol consumption via the AUDIT [37], (2) stage of change via the Readiness to Change Questionnaire–Treatment Version (RCQ-TV) [38], and (3) psychological distress via the K-10 [39]. These measures are detailed in the *Measures* section.

Once completed, individuals receive immediate personalized feedback via a “Personal Snapshot Report” (Multimedia Appendix 1) containing four major elements: (1) a summary of their results on each of the validated measures with normative data on alcohol consumption and psychological distress based on the Australian population, (2) psychoeducation on clinical guidelines (including country-specific information where available) and standard drink size, (3) harm reduction strategies, and (4) treatment suggestions (a range of digital and nondigital options are presented as part of a stepped-care approach based on their identified alcohol consumption risk level, ranging from self-management on the web to phone and face-to-face services, as well as mental health service information) and links to further information (information packs on alcohol-related topics, harm reduction information, information on withdrawal symptoms and detoxification, and how to access a psychologist via the GP). Although the information provided was largely Australian centric, where possible, additional information (eg, alcohol consumption guidelines for other countries and services available to overseas residents) was provided based on the participants’ location internationally.

Hyperlinks embedded within the Personal Snapshot Report allowed for the tracking of clicks on support and information links (“immediate online help-seeking”). Information packs were developed using evidence-based content by clinical psychologists on alcohol-related topics (managing urges, relapse prevention, mental health, sleep issues, and relationship issues), and these were linked to in a section made available only to A&WS participants on the Hello Sunday Morning website (with their use then quantified as part of “immediate online help-seeking”). Regarding information packs, clicks on app store links (Android and Apple) to download one of Hello Sunday Morning’s key digital health offerings—the Daybreak app—were also recorded as part of “immediate online help-seeking.”

### Measures

#### AUDIT Measure

The 10-item AUDIT [37] is considered the gold-standard measure to assess risk of alcohol-related harm, conceptualizing risk across 3 domains: alcohol consumption, dependence, and alcohol-related consequences. Scores range from 0 to 40, with higher scores indicating a greater likelihood of hazardous and harmful drinking. Australian norms are available [40]. As the AUDIT is based on a 12-month reference period, the AUDIT Consumption subscale (AUDIT-C) [41], comprising the first 3 questions of the AUDIT, is recommended as the outcome measure to assess changes over time. Previous research has shown that the AUDIT-C can predict clinical outcomes at 12 months [42].

#### K-10 Measure

The K-10 is widely used as a brief measure of psychological distress. Total severity scores range from 10 to 50, with higher scores indicating greater levels of psychological distress. The K-10 is the recommended screening measure for comorbid mental health conditions in individuals presenting for alcohol use disorder [43]. The temporal stability of the K-10 in treatment- and non–treatment-seeking samples has recently been confirmed [44], supporting its use as an outcome measure. The K-10 has demonstrated strong psychometric properties and can be used as a valid predictor of mental health, mood, and anxiety disorders [45-47]. Batterham et al [48] investigated the psychometric properties of 8 commonly used distress measures against an Australian population sample, and the K-10 demonstrated good predictive validity for a range of Diagnostic and Statistical Manual of Mental Disorders, Fifth Edition, disorders. Australian norms are available from the 2019 National Drug Strategy Household Survey [3].

#### RCQ-TV Measure

The RCQ-TV (revised) [38] is a 12-item measure that categorizes 3 possible stages of change: precontemplation, contemplation, and action. It is based on the transtheoretical model of behavior change [49,50] and asks about beliefs and assumptions to assess the stage of change for both reducing and quitting drinking. This measure was developed for use in conjunction with brief interventions among hazardous drinkers in different settings. Predictive validity in clinical samples has been established [51], and construct validity supporting the 3-factor structure (precontemplation, contemplation, and action) has been reported [52-54]. The RCQ-TV was recently validated in a non–treatment-seeking population [55].

#### Feedback and Follow-Ups

Feedback questions assessed (1) satisfaction with the A&WS and its components and likelihood of recommending the tool to others, (2) reasons for accessing the A&WS and previous use of any services and resources for alcohol- and mental health–related concerns, (3) further details regarding alcohol consumption patterns over the previous month (eg, number of drinking days during a typical week and high-consumption episodes), (4) barriers to changing drinking behavior and level of importance and confidence that changes to drinking behavior can be made, and (5) help-seeking intentions. Follow-ups comprised the AUDIT-C and K-10, RCQ-TV (3-mo follow-up only), alcohol consumption patterns over the previous month, and self-reported uptake of any resources or services.

### Statistical Analyses

Statistical analyses were conducted using SPSS Statistics for Windows (version 29.0; IBM Corp). Descriptive statistics were used to quantify respondent characteristics. Student 2-tailed *t* tests were used to compare continuous variables, whereas the Pearson chi-square test was used to compare categorical variables between 2 groups. The McNemar test was conducted to compare proportions of respondents (repeated measures) for 2 time points, and the Cochran Q was conducted to compare proportions of respondents (repeated measures) for >2 time points (with the McNemar test for post hoc testing in case of significant differences). Repeated-measure ANOVA was used to evaluate primary outcomes (continuous variables) with Bonferroni-corrected pairwise comparisons. Where the data violated sphericity assumptions, the Huynh-Feldt correction was applied. An a priori power analysis was conducted for outcomes at 3 months using G*Power [56] to determine the minimum sample size. The results indicated that the required sample size to achieve 80% power for detecting a small effect (0.01) at a significance criterion of .05 was 127 for repeated-measure ANOVA.

The number needed to screen (NNS) was calculated according to the method adopted by Whitton et al [57], which aligns with those used in multiarm clinical trials as described by Rembold [58]. As overviewed by Whitton et al [57], the NNS is derived from the number needed to treat statistic reported in clinical trials, reflecting the number of people who need to be screened to prevent 1 adverse event. It incorporates information about the prevalence of undetected diseases that may be identified via screening and is an indicator of the effectiveness of the screening program. As per Whitton et al [57], the NNS was calculated by computing the absolute risk (AR) of identifying undiagnosed symptoms (in this case, “moderate/high risk/very high risk” AUDIT scores and K-10 scores) through screening and the AR of identifying undiagnosed symptoms under a hypothetical *no screening* condition. Similarly, we assumed that, in the hypothetical *no screening* condition, no individuals with undiagnosed symptoms would have been identified who would not have already been identified through care as usual without screening. We then computed the difference in AR between these 2 conditions (ie, the AR deduction [ARD]) and, from that, calculated the NNS (which is the inverse of the ARD, ie, 1/ARD) [58].

### Ethical Considerations

The study protocol and consent procedures were approved by the Bellberry Human Research Ethics Committee (2022-01-031). Study participants provided informed consent on the web and were advised that participation was voluntary, that they were free to withdraw at any time, and that their data would be stored securely and anonymously.

## Results

### Participant Flow

**Figure 1:**
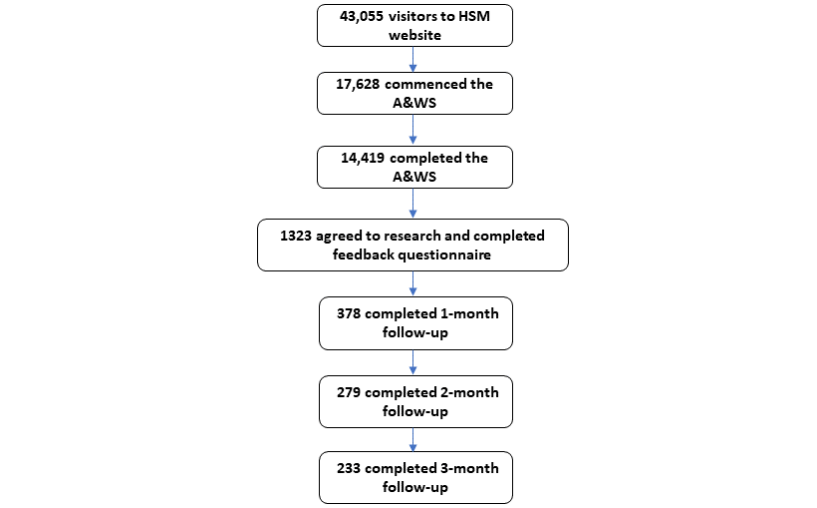
Participant flow. A&WS: Alcohol and Wellbeing Self-assessment; HSM: Hello Sunday Morning.

Of the 43,055 visitors to the Hello Sunday Morning website home page during the study period, 17,628 (40.94%) commenced the A&WS, and of these, 14,419 (81.8%) completed it. Of those 14,419 who completed the A&WS, 1323 (9.18%) agreed to participate in the follow-up research (providing complete feedback on the A&WS and their email address for ongoing follow-up assessments). The 1-, 2-, and 3-month follow-ups were completed by 28.57% (378/1323), 21.09% (279/1323), and 17.61% (233/1323) of the participants, respectively. Figure 1 shows the participant flow.

### Baseline Data

#### Overview

The characteristics of those who completed the A&WS (14,419/17,628, 81.8%) are presented in Table 1. Female individuals were overrepresented (8844/14,419, 61.34%), as were those residing in Australia (11,299/14,419, 78.36%). The average age of the sample was 49.9 (SD 12.7) years. The average AUDIT and K-10 scores were 15.0 (SD 8.1) and 20.7 (SD 8.0), respectively, indicating likelihood of alcohol dependence (moderate to severe alcohol use disorder) and mild mental health disorder. Just under one-third (4331/14,419, 30.04%) of the respondents were in the “Action” stage of change.

**Table 1:** Overall respondent characteristics (N=14,419).

				Values
Female individuals, n (%)				8844 (61.34)
**Country of residence, n (%)**				
	Australia		11,299 (78.36)	
	United States		1453 (10.08)	
	United Kingdom		674 (4.67)	
	Canada		217 (1.5)	
	New Zealand		277 (1.92)	
	Other		499 (3.46)	
**Alcohol consumption**				
	**AUDIT^a^ score, mean (SD)**		15.0 (8.1)	
		Low risk, n (%)	3040 (21.08)	
		Moderate risk, n (%)	4820 (33.43)	
		High risk, n (%)	2275 (15.78)	
		Very high risk, n (%)	4284 (29.71)	
**Psychological distress**				
	**K-10^b^ score, mean (SD)**		20.7 (8.0)	
		Low risk, n (%)	4687 (32.51)	
		Moderate risk, n (%)	3966 (27.51)	
		High risk, n (%)	3453 (23.95)	
		Very high risk, n (%)	2313 (16.04)	
**Stage of change (RCQ-TV^c^), n (%)**				
	Precontemplation		2370 (16.44)	
	Contemplation		7718 (53.53)	
	Action		4331 (30.04)	

^a^AUDIT: Alcohol Use Disorders Identification Test.

^b^K-10: Kessler Psychological Distress Scale.

^c^RCQ-TV: Readiness to Change Questionnaire–Treatment Version.

#### Follow-Up Research Participant Subsample

Regarding those who went on to complete the feedback and research questions following completion of the A&WS (n=1323), female individuals were again overrepresented (907/1323, 68.56%), as were those residing in Australia (976/1323, 73.77%). Within this subsample, participants were significantly older (mean age 50.8, SD 12.0 y vs 49.9, SD 12.8 y; *t_14417_*=2.5; *P*=.01) and had higher mean AUDIT (18.3, SD 8.3 vs 14.6, SD 8.0; *t_14417_*=15.8; *P*<.001) and K-10 (22.9, SD 8.4 vs 20.5, SD 8.0; *t_14417_*=10.4; *P*<.001) scores than those who only completed the A&WS. In terms of stage of change, a comparable proportion of participants were in the “Action” phase (375/1323, 28.34% vs 4331/14,419, 30.04%; *χ*_1_^2^=1.9; *P*=.17).

Typical alcohol consumption patterns over the previous month were assessed in this group. Most (1253/1323, 94.71%) consumed at least one drink containing alcohol. During a typical week, the average number of drinking days was 4.72 (SD 2.2), and on a typical drinking day, the average number of standard drinks consumed was 7.1 (SD 7.0). High-consumption episodes were common—the largest number of standard drinks consumed in a single day on average was 11.1 (SD 8.1), and binge drinking episodes (defined as ≥4 standard drinks in a single day) occurred weekly for 29.1% (385/1323) of the participants and daily or almost daily for 29.33% (388/1323) of the participants.

#### Reasons for Accessing the A&WS and Use of Previous Services and Resources

##### Overview

Research subsample participants were presented with a series of statements to describe what prompted them to access the A&WS as well as previous services or resources accessed for alcohol-related concerns. Participants were invited to select any that applied (Table 2).

**Table 2:** Prompts to access the Alcohol and Wellbeing Self-assessment (A&WS) and use of previous services and resources (N=1323).

		Participants, n (%)
**Reasons for accessing the A&WS**		
	I wanted to assess my drinking out of curiosity	349 (26.38)
	I’ve realized there is an issue with my drinking and I want to know more	541 (40.89)
	I’m looking for some information on how to make changes to my drinking	546 (41.27)
	I am ready to make changes to my drinking and need some support	687 (51.93)
	I am having other difficulties that are impacting on my drinking	145 (10.96)
	A friend or family member expressed concerns about my drinking	179 (13.53)
	A health professional expressed concerns about my drinking	114 (8.62)
	Other (please specify)	58 (4.38)
**Previous services and resources used for alcohol-related concerns**		
	GP^a^ or physician	292 (22.07)
	Psychologist, psychiatrist, and other mental health professional	262 (19.8)
	SMART^b^ recovery	37 (2.8)
	AA^c^	124 (9.37)
	Face-to-face alcohol support services	95 (7.18)
	Daybreak app	196 (14.81)
	Online support service or alcohol reduction mobile apps	131 (9.9)
	Phone helplines	27 (2.04)
	Reading articles and books about alcohol use	416 (31.44)
	Self-monitoring alcohol consumption	443 (33.48)
	Other	56 (4.23)
	None of the above	455 (34.39)

^a^GP: general practitioner.

^b^SMART: Self-Management and Recovery Training.

^c^AA: Alcoholics Anonymous.

The most common reasons for accessing the A&WS included (1) being ready to make changes to their drinking and needing support, (2) looking for information on how to make changes to their drinking, and (3) realizing that they had an issue with drinking and wanting to know more. Concerns expressed by others (eg, family, friends, and health professionals) with regard to drinking were the least common reasons prompting participants to access the A&WS.

In terms of previous services and resources ever used for alcohol-related concerns, self-monitoring of alcohol consumption and reading books or articles about alcohol use were the most common. Approximately 1 in 5 participants had consulted a health professional (eg, GP or physician or psychologist, psychiatrist, or other mental health professional). One-third (455/1323, 34.39%) had not accessed any of the services or resources listed for alcohol-related concerns.

When specifically asked whether they had ever previously attended GP or physician appointments for alcohol use reasons (yes or no response), 20.86% (276/1323) endorsed this. Similarly, when asked about attendance for mental health reasons, 59.11% (782/1323) answered affirmatively.

##### Alcohol Use Issues: Number Needed to Treat

Of the 1323 participants, 910 (68.78%) who were previously unidentified or untreated scored in the “moderate/high risk/very high risk” group on the AUDIT, yielding an AR_screening_ of 910/1323=0.6878. Under the hypothetical *no screening* condition, we assumed that none of these respondents would have been detected, yielding a no AR_screening_ of 0/1323=0.

The ARD was calculated as AR_screening_ – AR_no screening_ (910/1323 – 0/1323=0.6878).

The NNS (the inverse of the ARD) was calculated as 1/0.6878=1.45. This indicates that, for every 2 patients who are offered alcohol screening, 1 individual with previously unidentified or untreated alcohol use issues will be identified.

##### Mental Health Issues: Number Needed to Treat

Of the 1323 respondents, 340 (25.7%) who were previously unidentified or untreated scored in the “moderate/high risk/very high risk” group on the K-10. As in the previous case, the NNS was quantified as 1/0.25699=3.89. This indicates that, for every 4 patients who are offered mental health screening (within the context of alcohol screening), 1 individual with previously unidentified or untreated alcohol use issues will be identified.

#### Alcohol Behavior Change Goals

Participants (n=1323) were asked about their main alcohol behavior change goals, along with a list of common barriers to changing drinking behavior (as per Han et al [59]). Goal-wise, most sought to reduce their drinking (770/1323, 58.2%) or quit drinking altogether (415/1323, 31.37%). The remainder wanted to maintain their current achievements (75/1323, 5.67%) or learn more about alcohol (36/1323, 2.72%). The top 3 selected barriers to changing drinking behavior were “Alcohol is part of my culture” (713/1323, 53.89%), “My friends and family drink*”* (672/1323, 50.79%), and “I can’t say no” (565/1323, 42.71%).

Finally, participants were asked to rate how important (0=not important at all; 10=extremely important) it was to them to change their drinking and their level of confidence (0=not confident at all; 10=extremely confident) to make this change. Mean scores for importance and confidence were 8.0 (SD 2.7) and 5.6 (SD 2.8), respectively.

#### Feedback on A&WS

Participants were presented with a series of statements with regard to components of the A&WS and asked about the extent to which they were satisfied (5-point scale: *very unsatisfied* to *very satisfied*) or agreed (5-point scale: *strongly disagree* to *strongly agree*) with each statement (Table 3).

Most (1038/1323, 78.46%) were satisfied or very satisfied with the A&WS overall and the Personal Snapshot Report, with most (1265/1323, 95.62%) finding it easy to complete. The information in the Personal Snapshot Report was considered of high utility by most, with 82.62% (1093/1323) indicating that the feedback was useful and 78.84% (1043/1323) indicating that the services and resources information was useful. Although a lesser proportion (850/1323, 64.25%) agreed with the statement that the experience of completing the A&WS had helped them learn something new about themselves, many (1011/1323, 76.42%) agreed that this had prompted them to think about the next steps to change their relationship with alcohol.

Participants (n=1323) were asked how likely they would be to recommend the A&WS to others (0=very unlikely to recommend; 10=very likely to recommend). The mean score was 7.0 (SD 2.8).

**Table 3:** Satisfaction (satisfied or very satisfied) and agreement levels (agree or strongly agree) in relation to Alcohol and Wellbeing Self-assessment (A&WS) components (N=1323).

	Participants, n (%)
Overall, how satisfied are you with the A&WS	1038 (78.46)
Overall, how satisfied are you with the Personal Snapshot Report	1046 (79.06)
I found the A&WS easy to complete	1265 (95.62)
I found the Personal Snapshot Report results feedback useful	1093 (82.62)
I found the information provided in the Personal Snapshot Report (resources and services) useful	1043 (78.84)
Overall, this experience has helped me learn something new about myself	850 (64.25)
Overall, this experience has got me thinking about the next steps I can take to change my relationship with alcohol	1011 (76.42)

#### Help-Seeking Intentions

Participants were presented with a list of options (Table 4) and asked which of them they planned to do next now that they had completed the A&WS.

The top 3 help-seeking intentions were to check out some of the suggested web-based resources and information (743/1323, 56.16%), download the Daybreak app (641/1323, 48.45%), and talk to a trusted family member or friend (257/1323, 19.43%). Less than 1 in 5 indicated that they would go on to access formal support services (eg, hotlines, web-based services, or making an appointment with a health professional).

**Table 4:** Help-seeking intentions following completion of the Alcohol and Wellbeing Self-assessment (N=1323).

	Participants, n (%)
Check out some of the online resources and information suggested	743 (56.16)
Get in touch with some of the services recommended (eg, hotlines and online services)	232 (17.54)
Download the Daybreak app	641 (48.45)
Make an appointment with my GP^a^, physician, or health care provider	133 (10.05)
Talk to someone I trust (eg, family and friends)	257 (19.43)
Other	116 (8.77)
None of the above	162 (12.24)

^a^GP: general practitioner.

### Follow-Up Data

#### Overview

One-way repeated-measure ANOVA was conducted to evaluate changes over time (from baseline to 3 mo) following completion of the A&WS in terms of alcohol consumption (AUDIT-C scores and typical consumption patterns), psychological distress (K-10 scores), and confidence scores. Stage of change groups (based on RCQ-TV scores) were also examined using the McNemar test to compare the proportion of participants in the precontemplation or contemplation versus action phase from baseline to 3 months. The Mauchly test indicated that the data for all alcohol consumption indicators (AUDIT-C scores, number of drinking days during a typical week, number of standard drinks on a typical drinking day, and the largest number of standard drinks in a single episode) violated the assumption of sphericity; therefore, *df* were corrected using Huynh-Feldt estimates of sphericity (*χ*^2^_5_=49.5; *P*<.001; ε=0.8; *χ*^2^_5_=45.1; *P*<.001; ε=0.8; *χ*^2^_5_=163.6; *P*<.001; ε=0.6; *χ*^2^_5_=128.0; *P*<.001; ε=0.6).

#### Alcohol Consumption

**Figure 2:**
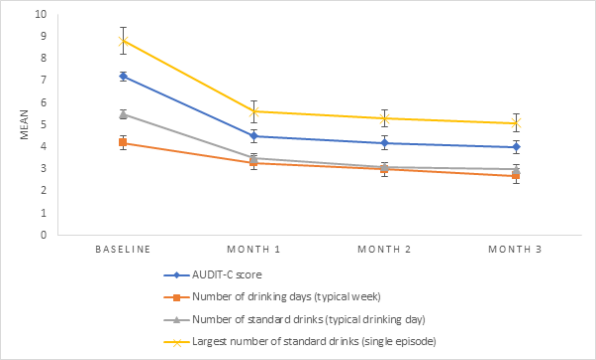
Estimated marginal means (alcohol consumption). AUDIT-C: Alcohol Use Disorders Identification Test Consumption subscale.

A total of 10.81% (143/1323) of the participants completed the AUDIT-C at all time points (baseline and months 1-3). AUDIT-C scores significantly decreased over time (*F*_2.5,356.1_=63.3; *P*<.001). Follow-up comparisons indicated that each pairwise difference was significant from baseline to 3 months (*P*<.001 in all cases; baseline mean 7.2, SE 0.3; month 1 mean 4.5, SE 0.3; month 2 mean 4.2, SE 0.3; month 3 mean 4.0, SE 0.3; Figure 2).

A total of 10.73% (142/1323) of the participants provided data on *number of drinking days during a typical week* at all time points. The number of drinking days during a typical week decreased significantly over time (*F*_2.5,353.5_=23.5; *P*<.001). Follow-up comparisons indicated that each pairwise difference was significant from baseline to 3 months (*P*<.001 in all cases; baseline mean 4.2, SE 0.2; month 1 mean 3.3, SE 0.2; month 2 mean 3.0, SE 0.2; month 3 mean 2.7, SE 0.2; Figure 2).

A total of 10.73% (142/1323) of the participants provided data on *number of standard drinks on a typical drinking day* at all time points. The number of standard drinks consumed on a typical drinking day decreased significantly over time (*F*_1.9,244.4_=18.9; *P*<.001). Follow-up comparisons indicated that each pairwise difference was significant from baseline to 3 months (*P*<.001 in all cases; baseline mean 5.5, SE 0.5; month 1 mean 3.5, SE 0.3; month 2 mean 3.1, SE 0.3; month 3 mean 3.0, SE 0.3; Figure 2).

A total of 10.58% (140/1323) of the participants provided data on *largest number of standard drinks consumed in a single episode* at all time points. The largest number of standard drinks consumed in a single episode decreased significantly over time (*F*_1.8,261.6_=22.0; *P*<.001). Follow-up comparisons indicated that each pairwise difference was significant from baseline to 3 months (*P*<.001 in all cases; baseline mean 8.8, SE 0.6; month 1 mean 5.8, SE 0.5; month 2 mean 5.2, SE 0.4; month 3 mean 5.1, SE 0.4; Figure 2).

A total of 10.66% (141/1323) of the participants provided data on *≥4 standard drinks in a single day (ie, binge drinking episodes)* at all time points. The Cochran Q test indicated that there was a statistically significant reduction in the proportion of binge drinking episodes over time (*χ*^2^_3_=55.8; *P*<.001). McNemar post hoc testing (Bonferroni corrected) revealed significantly reduced binge drinking episodes from baseline to each time point (*P*<.001 in all cases; baseline: 64.9%; month 1: 56%; month 2: 53.2%; month 3: 50.3%).

#### Psychological Distress

**Figure 3:**
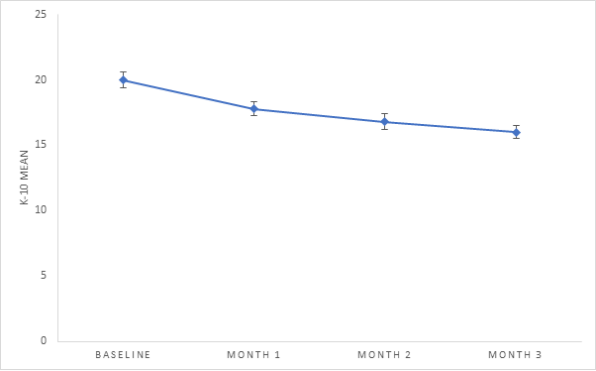
Estimated marginal means (psychological distress). K-10: Kessler Psychological Distress Scale.

A total of 10.51% (139/1323) of the participants completed the K-10 at all time points. The Mauchly test indicated that the assumption of sphericity was violated (*χ*^2^_5_=33.4; *P*<.001); therefore, *df* were corrected using Huynh-Feldt estimates of sphericity (ε=0.9). K-10 scores decreased significantly over time (*F*_2.6,366.0_=31.5; *P*<.001). Follow-up comparisons indicated that each pairwise difference was significant from baseline to 3 months (*P*<.001; baseline mean 20.0, SE 0.6; month 1 mean 17.8, SE 0.5; month 2 mean 16.8, SE 0.6; month 3 mean 15.8, SE 0.5; Figure 3).

#### Confidence

A total of 10.43% (138/1323) of the participants provided data on confidence scores at all time points. The Mauchly test indicated that the assumption of sphericity was violated (*χ*^2^_5_=16.3; *P*=.006); therefore, *df* were corrected using Huynh-Feldt estimates of sphericity (ε=0.9). Confidence scores increased significantly over time (*F*_2.8,386.9_=8.9; *P*<.001). Follow-up comparisons indicated that pairwise differences were significant from baseline to 2 (*P*=.008) and 3 (*P*<.001) months, with a nonsignificant upward trend from baseline to 1 month (*P*=.07; baseline mean 6.2, SE 0.2; month 1 mean 6.9, SE 0.2; month 2 mean 7.0, SE 0.2; month 3 mean 7.4, SE 0.2).

#### Stage of Change

The McNemar test was conducted to compare the proportion of respondents in the precontemplation or contemplation phase and the action phase from baseline to 3 months. Of the 17.61% (233/1323) of participants who completed the RCQ-TV at both time points, a significantly higher proportion shifted from the precontemplation or contemplation phase to the action phase from baseline to the 3-month follow-up (90/233, 38.6% vs 144/233, 61.8%; *P*<.001).

#### Help Seeking

##### Immediate Web-Based Help Seeking

Of the 14,419 respondents who completed the A&WS, 658 (4.56%) accessed alcohol-related service links (eg, Daybreak app and National Alcohol and Other Drug Hotline), 144 (1%) accessed mental health–related service links (eg, MyCompass and Beyond Blue), and 786 (5.45%) accessed information links (eg, information packs on alcohol-related issues and national guidelines for alcohol consumption).

##### Self-Reported Help Seeking

Within the research subsample, service and resource use for alcohol-related concerns following completion of the A&WS was examined. The same list of services and resources presented at baseline was presented at each follow-up point. Table 5 outlines the self-reported uptake of the listed options at any time within the following 3 months. Data were available for 39.15% (518/1323) of the participants (Table 5).

**Table 5:** Self-reported service and resource use for alcohol-related concerns since completing the Alcohol and Wellbeing Self-assessment (N=518).

		Participants, n (%)
**Services and resources used for alcohol-related concerns**		
	GP^a^ or physician	59 (11.4)
	Psychologist, psychiatrist, or other mental health professional	60 (11.6)
	SMART^b^ recovery	4 (0.8)
	AA^c^	14 (2.7)
	Face-to-face alcohol support services	23 (4.4)
	Daybreak app	103 (19.9)
	Online support service or alcohol reduction mobile apps	70 (13.5)
	Phone helplines	8 (1.5)
	Reading articles and books about alcohol use	197 (38)
	Self-monitoring alcohol consumption	244 (47.1)
	Other	37 (7.1)
	None of the above	210 (40.5)

^a^GP: general practitioner.

^b^SMART: Self-Management and Recovery Training.

^c^AA: Alcoholics Anonymous.

Almost 60% of the participants (309/518, 59.7%) reported the use of at least 1 of the listed services and resources for alcohol-related concerns within 3 months of completing the A&WS. The top 3 included ongoing self-monitoring of alcohol consumption (244/518, 47.1%), reading articles and books about alcohol use (197/518, 38%), and using the Daybreak app (103/518, 19.9%).

As asked at baseline, participants were asked again at each follow-up point whether they had specifically attended a GP for alcohol use reasons or for mental health reasons. Of the 389 participants who indicated that they had not attended a GP for alcohol use reasons at baseline and provided follow-up data, 50 (12.9%) went on to do so during the 3 months following completion of the A&WS. In terms of seeking assistance for mental health from a GP, of the 193 participants who indicated that they had not done so at baseline and who provided follow-up data, 47 (24.4%) went on to do so during the 3 months following completion of the A&WS.

## Discussion

### Principal Findings

This study investigated the feasibility, acceptability, and clinical outcomes of participants who completed a web-based brief intervention seeking to help individuals change their relationship with alcohol. The key findings are now summarized.

Feasibility was demonstrated, with 17,628 individuals commencing the A&WS over a 9-month period and most (n=14,419, 81.8%) subsequently completing the tool. A high uptake suggests a level of concern or curiosity within the general community (both in Australia and internationally) regarding alcohol use. This, in addition to the minimal marketing undertaken to promote the A&WS, highlights a clear demand for web-based screening tools for alcohol use.

In terms of participants’ characteristics, almost 80% of those completing the A&WS (11,379/14,419, 78.92%) were in the moderate to very high risk range on the AUDIT, whereas almost 70% (9732/14,419, 67.49%) reported moderate to very high levels of psychological distress. As noted by Han et al [59], although web-based self-screening tools for alcohol use are likely to attract those who already have concerns about their drinking habits, the high prevalence of unhealthy drinking in this sample was striking. Alcohol consumption and psychological distress scores were significantly higher in those who went on to participate in the follow-up research component, with most (almost 80%; 1031/1323, 77.93%; *P*<.001 for both AUDIT and K-10) indicating that they had never attended a GP or physician for alcohol use reasons. Stigma surrounding substance use is an ongoing major barrier to treatment seeking, with previous studies in adults with problematic alcohol and other drug (AOD) use affirming that negative public attitudes toward substance users discouraged them from accessing treatment [60,61]. Interestingly, a higher proportion (782/1323, 59.11%) of participants in our study had attended a GP or physician appointment for mental health reasons, suggesting a higher level of acceptability of seeking treatment for these reasons rather than for alcohol-related reasons. Indeed, public attitudes appear to be more negative toward those with AOD issues than those with mental health concerns, possibly in part because of the perception of personal responsibility involved in AOD use [61].

Motivations for accessing the A&WS were primarily centered on feeling ready to make changes to drinking behavior (and needing support to do so) and seeking information on how to make those changes. This, in addition to previous use of services and resources for alcohol-related concerns most commonly falling into the self-help category (eg, self-monitoring of alcohol consumption or reading books or articles about alcohol use), supports the role of self-management, which may then act as a conduit to formal assistance from a health professional if required. These results also highlight the need to provide individuals with easily accessible self-help tools (eg, alcohol trackers and web-based resources) to facilitate change. Consistent with this, self-reported intentions for help seeking following completion of the A&WS were primarily web-based self-help strategies, including checking out the web-based resources and information suggested by the tool and downloading the Daybreak app. Intentions to access formal services (eg, getting in touch with web-based services and making an appointment with a health professional) were least commonly nominated, suggesting a preference for engaging in self-management (at least as an initial strategy). These findings align with the Global Drug Survey [62], which found that, for those intending to reduce their drinking and wanting help to cut down, web-based self-help tools were most preferred by those in Australia, New Zealand, and the United Kingdom as a source of support. Indeed, as overviewed by Venegas et al [63], “natural recovery” or “self-change” is well documented in the literature, whereby a substantial number of individuals have been shown to recover from alcohol use disorders without formal treatment.

Regarding participants’ impressions of the A&WS, feedback suggested that acceptability was high, with approximately 80% (1038/1323, 78.46%) reporting high satisfaction levels overall, and the feedback and services or resources information provided were rated as useful. Almost all participants (1265/1323, 95.62%) found the A&WS easy to use, indicating that they would recommend the tool to others. Encouragingly, most (1011/1323, 76.42%) agreed that the tool had prompted them to think about the next steps to change their relationship with alcohol. There is evidence that just monitoring one’s own health data can prompt changes in behavior [64] and that alcohol consumption assessments alone can influence alcohol consumption, acting as brief interventions in and of themselves [65].

Some interesting patterns emerged in relation to actual help seeking following completion of the A&WS. Although immediate web-based help-seeking rates were low (up to 786/14,419, 5.45%) compared with other studies reporting rates of 26% to 60% in those screened for mental health [28], help seeking was self-reported by almost 60% (309/518, 59.65%) of participants within the 3 months following completion of the A&WS. Positively, of those who had never seen a GP for alcohol use reasons, 12.9% (50/389) went on to do so during the 3 months following completion of the A&WS. Similarly, almost one-quarter (47/193, 24.4%) sought assistance for mental health from a GP within 3 months of completing the A&WS despite never having done so before. Although these results cannot solely be attributed to the A&WS, they are encouraging given the low levels of service use in this population [63]. Initiating help seeking is a process that takes time and will differ between individuals—perceived need for treatment, readiness to change, stigma, mental health, alcohol use severity, access to resources, cost, and other characteristics (eg, gender and age) are likely to influence how, when, and whether next steps are taken. This highlights the importance of meeting the individual where they are on their alcohol behavior change journey—light-touch personalized digital health offerings may bridge the gap to formal treatment (if needed) at a later stage as part of a stepped-care approach. Indeed, a longitudinal study of 2000 Australians found that intention to seek help for substance use and mental health concerns predicted actual help seeking 2 years later [66], supporting the need for longer-term follow-up of actual help-seeking behaviors.

Several positive clinical outcomes were observed in participants who completed the A&WS. As hypothesized, significant reductions in AUDIT-C and K-10 scores were observed over the 3-month follow-up period. The average score changes on the AUDIT-C represented a shift from the “moderate” risk category to the “low” risk category. Furthermore, typical alcohol consumption patterns improved significantly, with a reduced number of (1) drinking days during a typical week (1.5 less on average) and (2) standard drinks on a typical drinking day (2.5 less on average). Improvements were also observed for high-consumption episodes, with a significantly lower number of standard drinks consumed in a single episode (3.7 less on average) and significantly fewer binge drinking episodes at 3 months (14.6% reduction). Although encouraging, in the absence of a control group, the observed benefits cannot be attributed to the A&WS and may simply represent a regression to the mean, particularly considering the higher AUDIT and K-10 scores in this subsample at baseline. Furthermore, the high attrition rates (with only 143/1323, 10.81% and 139/1323, 10.51% of participants completing the AUDIT and K10, respectively, at all time points over the 3-mo follow-up period) limit the generalizability of the findings. Despite these limitations, the positive outcomes observed are consistent with those of other studies suggesting that e-SBIs may be potentially successful tools to reduce alcohol consumption [13,16,18,20,21].

There are 2 other findings worth noting in relation to clinical outcomes. First, participants reported a significant increase in confidence in relation to their ability to make changes to their drinking during the 3 months following completion of the A&WS. Increased awareness (eg, via provision of personalized feedback and ongoing self-monitoring) and provision of information and resources may empower individuals to make changes, ultimately building their confidence to enact those changes. Importantly, confidence to change has been found to predict improved alcohol use outcomes over the longer term irrespective of treatment, age, gender, and dependence severity [67]. Second, shifts in stage of change were observed, with a higher proportion of respondents falling into the “action” stage based on their RCQ-TV scores at the 3-month follow-up. Taken together, these results suggest that participants not only experienced positive changes in terms of alcohol consumption behavior and associated psychological well-being but also changed their relationship with alcohol in terms of their beliefs, assumptions, and confidence levels to continue to make those changes. These more diverse goals are an important factor in long-term behavior change, particularly considering the chronic, relapsing nature of alcohol use disorders.

Although not a main aim of this study, the NNS was quantified for alcohol and mental health issues. As noted earlier, the NNS is indicative of how many individuals would need to be screened to prevent 1 adverse event and is one of the criteria with which the efficacy of a screening tool such as the A&WS can be determined. The lower the NNS, the more pivotal screening would be. In our sample, 2 individuals needed to be screened to prevent 1 adverse event in relation to alcohol use, and 4 individuals needed to be screened to prevent 1 adverse event in relation to mental health. Our results differ 4-fold from those quantified for mental health issues by Whitton et al [57], who reported an NNS of 16. It is important to note the differing contexts for screening, which may partly explain the discrepancy—the primary context for the study by Whitton et al [57] was mental health screening, whereas the A&WS primarily focused on screening for alcohol use issues (and, as such, is located on the Hello Sunday Morning website, whose key focus is on alcohol use). Nonetheless, the lower NNS in our context highlights the need for screening for comorbid mental health conditions in a sample that presents for potential alcohol use issues. Although these findings are notable, our results are interpreted with caution; however, as previous help-seeking questions (used to estimate the NNS) were only asked of those who agreed to the follow-up research, our results may not represent the NNS more broadly within the community.

### Strengths and Limitations

This study had several strengths. The A&WS is a clearly defined e-SBI, encompassing clinically validated tools to assess alcohol use, psychological distress, and stage of change; evidence-based BCTs; and stepped-care recommendations for services and resources to meet the individual where they are on their alcohol behavior change journey. To our knowledge, this study is the first to (1) incorporate stage of change assessment into an alcohol use–focused e-SBI as part of a broader change goal, (2) measure both immediate web-based help seeking and self-reported uptake of services and resources over time, and (3) quantify NNS for alcohol use issues in a community sample within the web-based screening context. Finally, feasibility was evidenced by high uptake, with a large sample size completing the tool within a relatively short study time frame.

Study limitations are now considered. As noted previously, the major limitation of this study was the absence of a control group; therefore, a range of uncontrolled factors (eg, the benefits of simply participating and naturalistic variation in psychological well-being) may confound the results and preclude any conclusions with respect to intervention effectiveness. Furthermore, given the higher AUDIT and K-10 scores in the subsample at baseline, changes in scores may simply represent a regression to the mean. Therefore, the true impact of the A&WS on outcomes can only be formally determined via an RCT. Given its clearly defined components, the A&WS could easily be subjected to RCT assessment to determine which components are “active ingredients” contributing to change. Nonetheless, our results support the role of screening in identifying opportunities to encourage help seeking (whether via self-management strategies or seeking assistance from health professionals), which may in turn reduce the burden on higher-intensity services. Second, although all data were self-reported and, therefore, subject to response biases (eg, social desirability bias), data collection from web-based self-reports has been regarded as reliable and preferred [68], particularly within an anonymous context. Third, attrition rates were high over the course of the 3-month follow-up period, introducing a clear selection bias. Although we adopted several engagement strategies (reminder emails and incentives), attrition rates are commonly high in eHealth studies [69], including studies evaluating the effectiveness of e-SBIs for reducing excessive drinking [70], where rates can be as high as 83.5%. Fourth, the research follow-up subsample was not representative of A&WS completers; therefore, the results cannot be generalized to broader community samples and require replication. Generalizability internationally is also limited as the study was heavily geared toward the Australian population. Finally, the follow-up time frame was relatively short, focusing on 3-month outcomes. Given the chronic, relapsing nature of alcohol use issues, longer-term follow-ups are necessary to determine whether the outcomes are sustained over time. The data from 6- and 12-month follow-ups from the A&WS will be reported in forthcoming publications.

### Conclusions

The free and publicly available A&WS is a highly feasible and acceptable tool that may empower individuals to make changes to their alcohol consumption and improve their psychological well-being. Web-based tools such as the A&WS have the potential to fill the treatment gap, reaching large numbers of adults who might not otherwise have received help. Further testing via an RCT, tailoring, and targeting will help position the A&WS as a freely available, scalable, and potentially effective tool complementing the AOD and mental health treatment ecosystem.

## Appendix

Multimedia Appendix 1 Personal snapshot report.

## Abbreviations

A&WS: Alcohol and Wellbeing Self-assessment
AOD: alcohol and other drug
AR: absolute risk
ARD: absolute risk deduction
AUDIT: Alcohol Use Disorders Identification Test
AUDIT-C: Alcohol Use Disorder Identification Test Consumption subscale
BCT: behavior change technique
e-SBI: electronic screening and brief intervention
GP: general practitioner
K-10: Kessler Psychological Distress Scale
NNS: number needed to screen
RCQ-TV: Readiness to Change Questionnaire–Treatment Version
RCT: randomized controlled trial
SBI: screening and brief intervention
