# Supplementary figures and images for: Preliminary Clinical Outcomes of the Hello Sunday Morning Alcohol and Wellbeing Self-Assessment: Feasibility and Acceptability Study
Source: JMIR Form Res. 2023 Oct 24;7:e48245. doi: 10.2196/48245 (PMC10630865; doi:10.2196/48245)

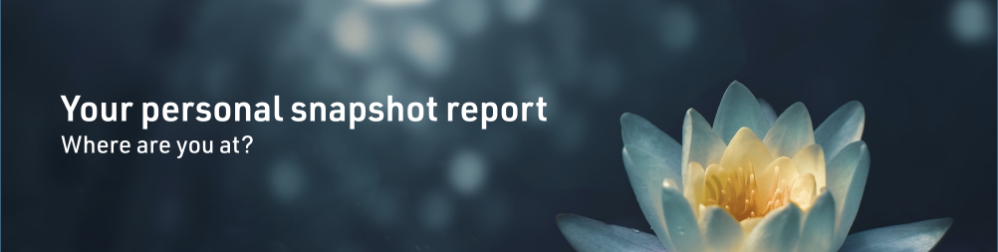

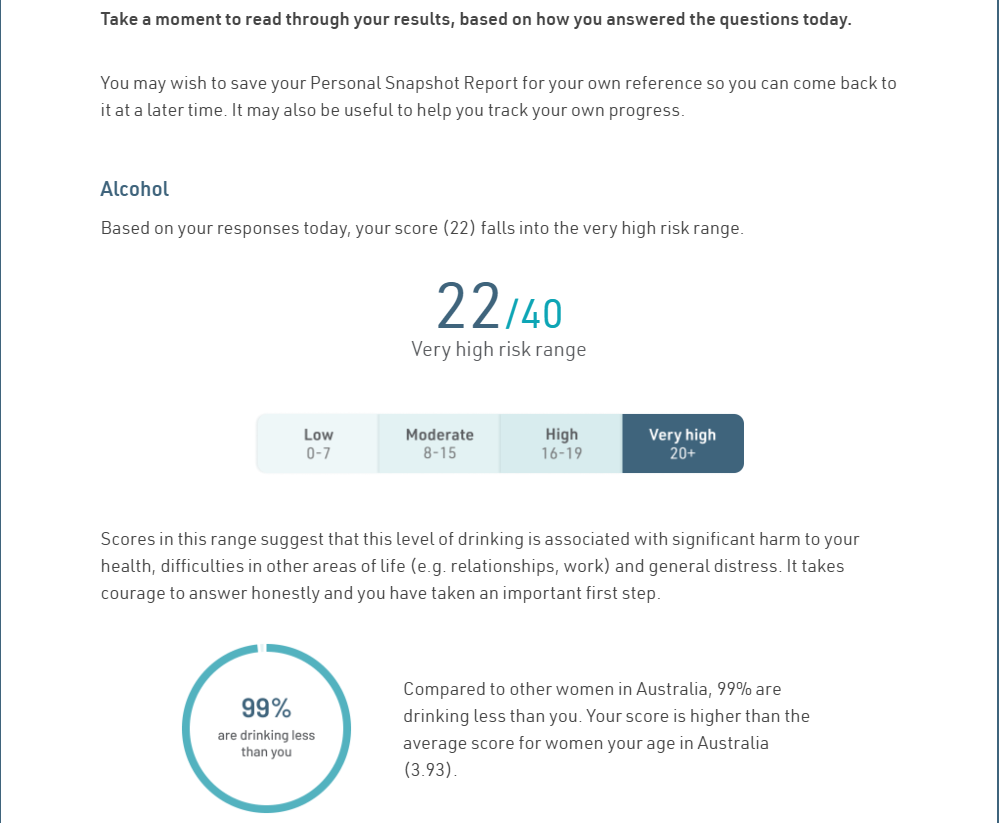

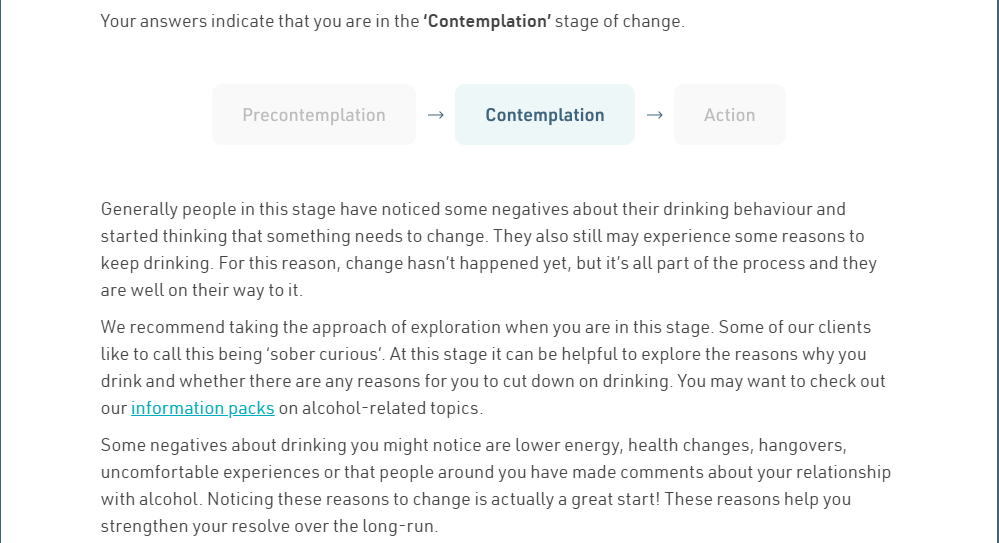

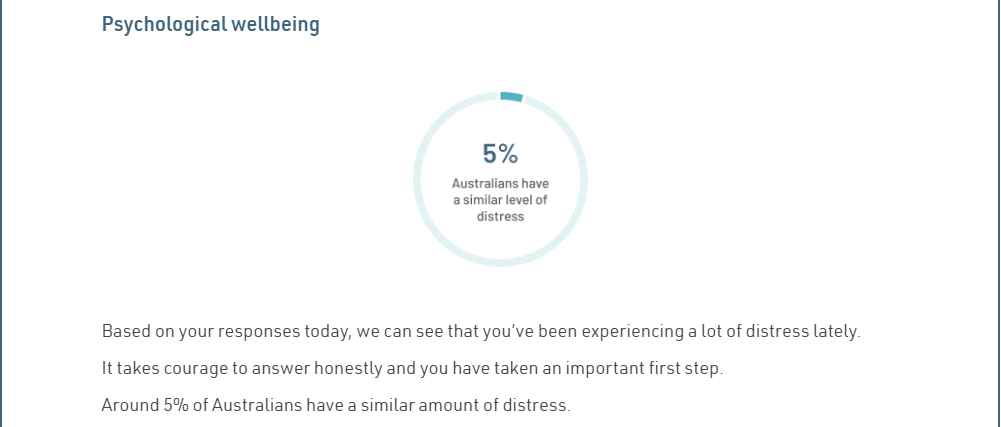

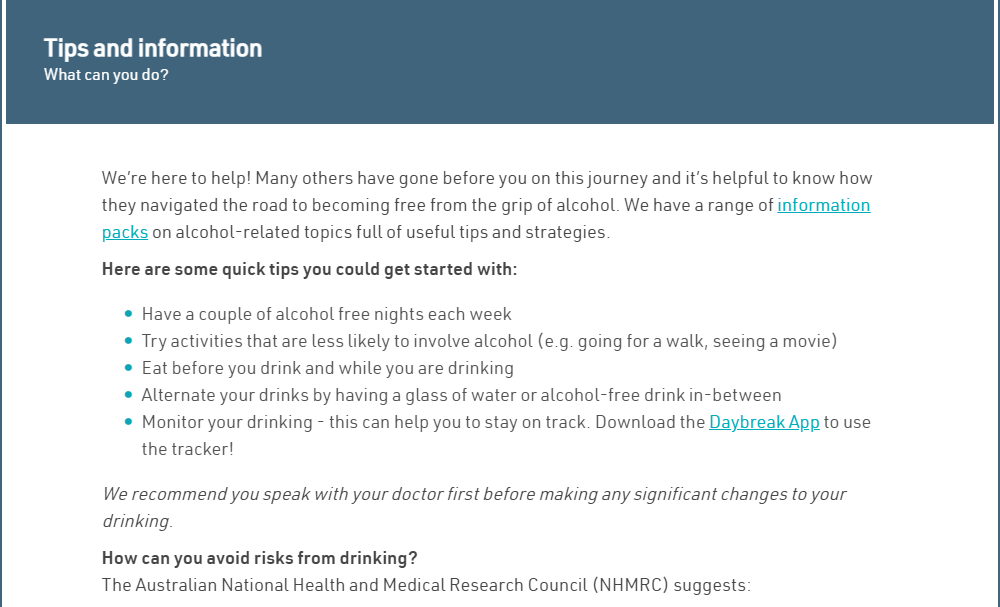

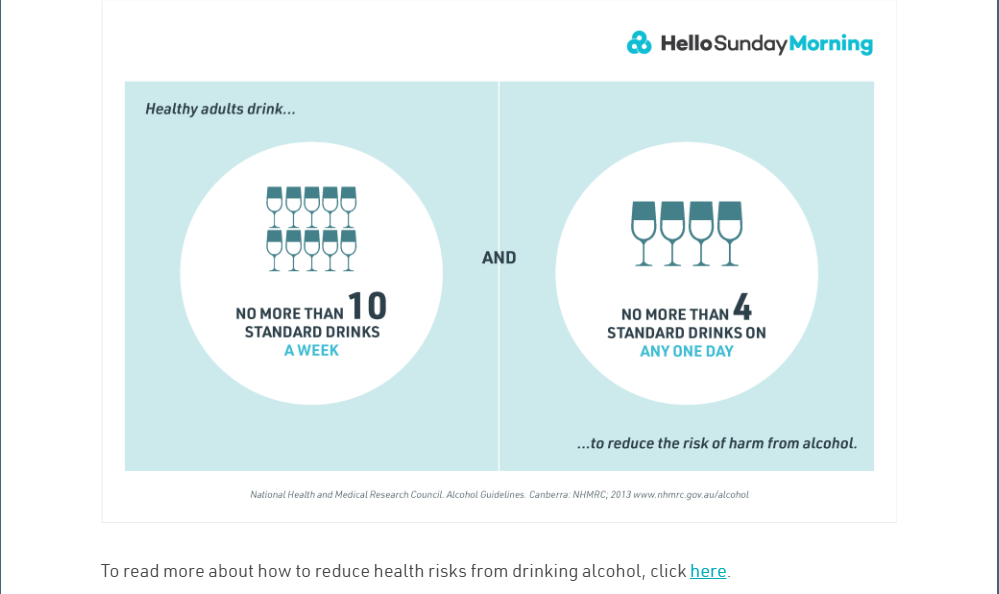

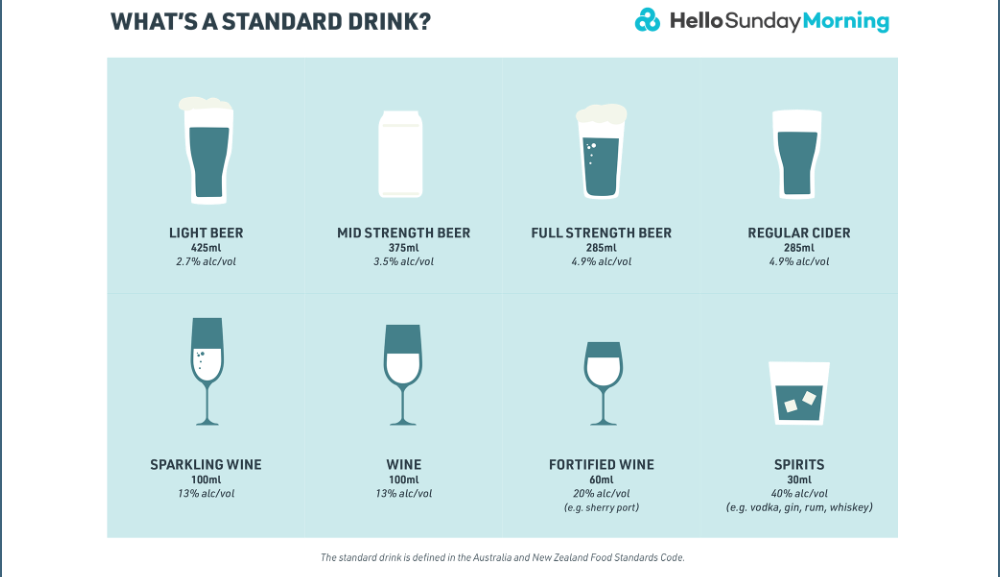

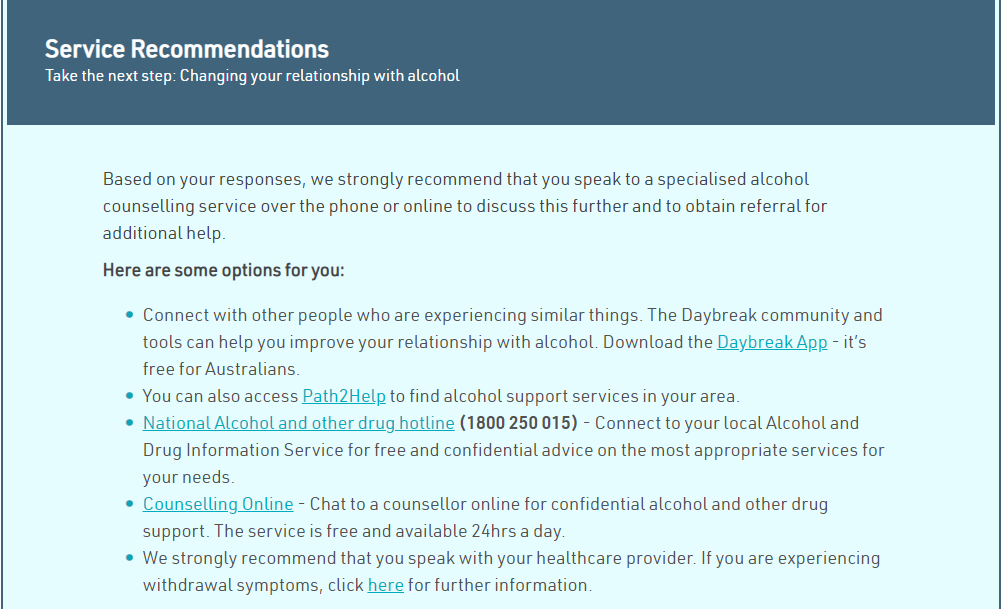

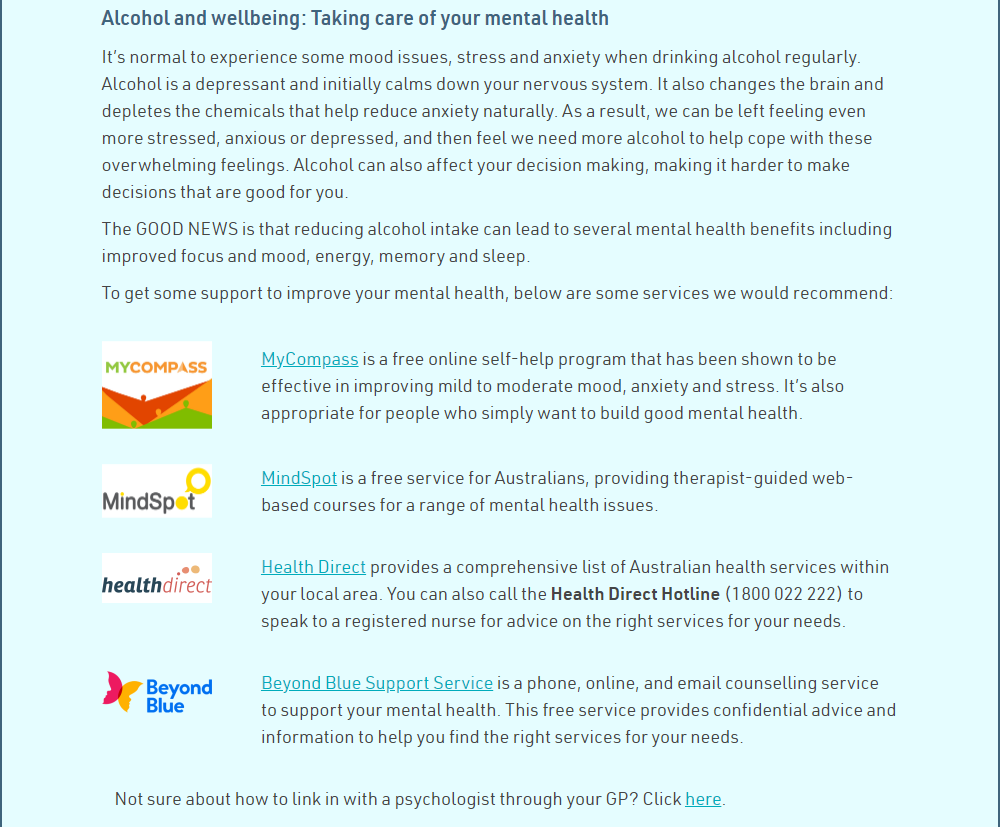

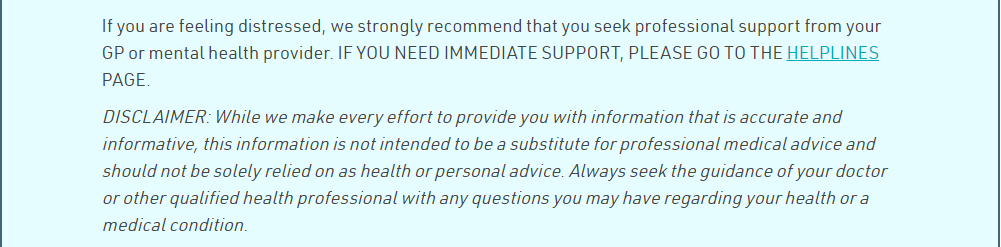

Supplement: Multimedia Appendix 1 [file formative_v7i1e48245_app1.docx]
